# Multifractality of the heartbeat dynamics after beating heart myocardial revascularization

**DOI:** 10.1186/1749-8090-8-S1-O109

**Published:** 2013-09-11

**Authors:** J Ksela, JM Kalisnik, V Avbelj, B Gersak

**Affiliations:** 1 Department of Cardiovascular Surgery, University Medical Center Ljubljana, Slovenia; 2 Department of Communication Systems, Jozef Stefan Institute, Ljubljana, Slovenia

## Background

Recent studies suggest that time series of healthy human interbeat intervals belong to a special class of bio-signals displaying multifractal properties. The breakdown of multifractality was observed in congestive heart failure and angina pectoris; however, there has been no attempt to evaluate multifractal behavior before and after beating heart myocardial revascularization (off-pump CABG).

## Methods

Sixty consecutive patients with isolated multivessel coronary artery disease scheduled for off-pump CABG were included in the study. Twenty-four hour Holter recordings were performed preoperatively and on the seventh postoperative day. Multifractal properties of the RR data set were determined for both, day- (12:00h to 18:00h) and night-time (00:00h to 06:00h) periods of the ECG recordings containing at least 95% of pure sinus rhythm. Multifractal spectrum τ at q=3 (τ(q=3)), the peak position of the singularity spectrum (h_top) and the width of the singularity spectrum (Δh) were calculated by wavelet modulus maxima method as proposed by Ivanov et al. Mean differences over time were tested using paired-samples t-test. Results are reported as mean ± SE; p<0.05 or less was considered significant.

## Results

Preoperatively, τ(q=3) was -0.52±0.18 and -0.49±0.17, h_top 0.20±0.07 and 0.15±0.07 and Δh 0.31±0.14 and 0.71±0.14 for day-time and night-time period, respectively. Postoperatively, τ(q=3) was significantly higher for day-time period (-0.43±0.23, p=0.015), whereas h_top and Δh were significanly higher for both, day- and night-time periods (0.25±0.07, p<0.001 and 0.19±0.06, p=0.002 for h_top and 0.41±0.20 and 0.31±0.19 for Δh, respectively).

## Conclusions

Significant postoperative increase of all multifractal parameters, except of τ(q=3) for night-time periods, clearly indicates that a marked breakdown of multifractal behavior into monofractal can be observed following off-pump CABG, indicating that multifractality is mostly governed by vagal activity.

